# Increasing Risks to the Health of the Invertebrates—Balancing between Harm and Benefit

**DOI:** 10.3390/ani14111584

**Published:** 2024-05-27

**Authors:** Tatiana V. Kuznetsova, Valentina A. Kudryavtseva, Larisa L. Kapranova

**Affiliations:** 1 St. Petersburg Federal Research Center of the Russian Academy of Sciences, 199178 St. Petersburg, Russia; valenkud@yandex.ru; 2 A.O. Kovalevsky Institute of Biology of the Southern Seas of the Russian Academy of Sciences, 299011 Sevastopol, Russia; lar_sa1980@mail.ru

**Keywords:** surfactants, sanitizers, heavy metals, biological effects, legislation and nature protection, non-invasive monitoring methods, invertebrate welfare

## Abstract

**Simple Summary:**

Emerging challenges associated with the COVID-19 (SARS-CoV-2) pandemic include the unpredictable biological effects of highly recommended detergents and disinfectants to reduce the risk of human infection. How these classes of substances affect living organisms, especially the health of the invertebrates as common representatives of biota, is still unclear. Due to the exceptional ability of disinfectants and detergents to penetrate biological membranes and alter the pH of the environment, these classes of substances, together with other environmental pollutants of concern (e.g., heavy metals), can have pronounced negative effects on macrobenthic invertebrates. The review analyzes the current state of research in this area and discusses ways to reduce risks to invertebrates.

**Abstract:**

The article discusses the issue of extensive use of detergents and sanitizers in the time of new challenges associated with the COVID-19 (SARS-CoV-2) pandemic. These agents could pose threats to the existence of free-living invertebrates as essential components of the ecosystem. The biological effects of the mentioned classes of substances, their metabolites, and combined effects in the mixture have not been studied enough. The main challenges in trying to balance the threats and benefits of using such substances are the lack of knowledge of the biological effects of these products, the gaps in testing invertebrates’ responses, and changes in environment-related regulations to minimize risks to animals and humans. Numerous studies in this field still leave research gaps, particularly concerning the combined toxicity of well-known and widely used disinfectants, surfactants, and heavy metals, posing potential future challenges. Additionally, the review identified the need for additional testing of invertebrates for their sensitivity to disinfectants and surfactants of different compositions, including improved (non-invasive) methods, studies for early life stages, and comparative studies of species resilience.

## 1. Introduction

The SARS-CoV-2 virus spread has brought new challenges not only to the current epidemiological situation, but also raised fears of threats to environmental safety [1,2]. Since the first months of recording the pandemic, the World Health Organization (WHO) and the Russian Federal Service for Supervision of Consumer Rights and Human Welfare Protection (Rospotrebnadzor) have recommended the use of disinfectants and sanitizers to prevent infection and spread of the disease in crowded places, including hospitals, transport, institutions, schools, and households [3,4].

The purpose of these agents is to combat microorganisms and viruses. The majority of products that enter the distribution network include ethyl or isopropyl alcohol, propylene glycol, triclosan, formic, and, occasionally, salicylic acid, in addition to various fragrances and other ingredients.

However, people who use sanitizers frequently complain about dry skin, peeling, occasional skin flushing, and shortness of breath after several days of use, which manifest the adverse effects of these agents on the human body.

While a large proportion of the substances used in detergent formulations are subject to compulsory registration within the regulation of the Registration, Evaluation, Authorisation, and Restriction of Chemicals (REACH), there is still often little or even nothing known about their environmental impact. In only a few cases they are included in water monitoring programs (e.g., benzotriazole).

Wastewater from hospitals may contain a mixture of different drugs, radionuclides, anionic, cationic and amphoteric/non-ionic surfactants, disinfectants, and pathogenic microorganisms. The problem with most drugs is that, even after ordinary treatment, they are still present in wastewater [5], violating water quality requirements prescribed in Directive 98/83/EC [6].

**Figure 1:**
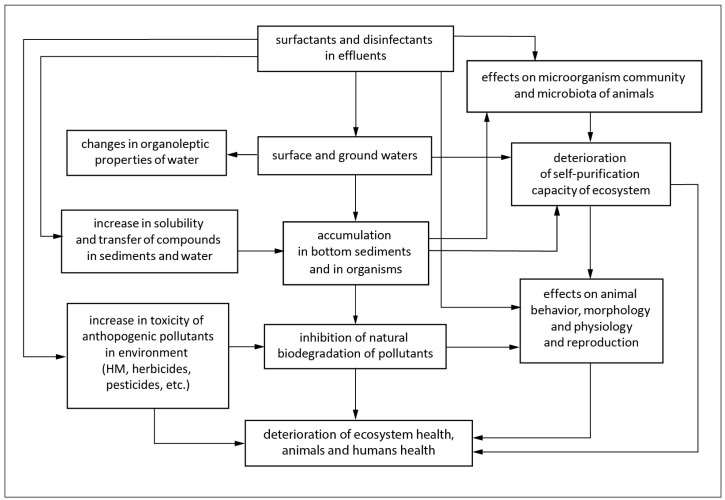
Common pathways of surfactant and disinfectant entry into the environment and the main consequences.

All of these substances and their metabolites are released to the environment, where they may further react and interact with other materials, pollutants, or their metabolites. Hospital wastewater, moreover, contributes to the high resistance of bacteria to antibiotics. This subsequently affects the long-term ecological balance under chronic exposure [7]. Wastewater treatment plants are typically not designed to remove these chemicals both from biologically treated wastewater and from sludge. Sanitizers and disinfectants are mostly concentrated in the sludge from these plants. Common pathways of the surfactant and disinfectant entry into the environment and the main targets are presented in the diagram (Figure 1).

Using simple chemical analysis, it is not possible to assess the complex interactions and synergy of these substances and their actual effects on biological systems, as well as the threats to the integrity of the ecosystem under impact, including its abiotic and biotic components. In these cases, biological tests provide reliable information about the effects of the mentioned materials on the quality of treated wastewater entering natural water bodies and their effects on ecosystems and human health.

At present, there are no particular technologies and techniques for removing these wastewater contaminants and their metabolites. As a result, the levels of disinfectants in wastewater, and subsequently in surface waters, including those used by fisheries, will rapidly increase.

To a larger extent, the accumulation of sanitizers and their metabolites in aquatic environments can disturb the safety of both terrestrial and aquatic flora and fauna. This situation undoubtedly raises concerns among environmental scientists, physicians, environmental agency experts, and the general public.

Thus, there is a pressing need for novel information to evaluate the possible hazards of negative impacts, which can often be delayed, on the health of animals and humans. This evaluation should take into consideration the potential combined effects of contaminants in complex aquatic systems like surface waters.

## 2. General Information on Disinfectants: Benefits and Harm

In all cases, US EPA (2023) recommends the use of surfactants of different origins and compositions before disinfection [5].

Disinfectants are defined as “antimicrobial and antiviral products” [8]. In different countries, Environmental Protection Agencies (EPA) regulate and register antimicrobial agents and ensure that these agents meet minimum requirements for the registration to certify that they are effective against certain types of microorganisms or viruses. The use of disinfectants (including sanitizers) and antiseptics, recommended by WHO, is projected to increase [1]. However, it still requires consideration of the balance of benefits (killing of dangerous viruses and microorganisms) and possible harmful effects (indirect environmental and health impacts).

On the other hand, disinfectants have been often and successfully used in agriculture and aquaculture. In aquaculture, they help fight not only microorganisms and viruses, but can also significantly reduce the number of parasites and reduce the number of fungal diseases in farmed fish, crustaceans, and mollusks (e.g., [7]).

The most common disinfectants are chlorine products in their various formulations and forms (solution, solid, or powdery material).

At the same time, elevated levels of chlorine have the potential to cause skin or mucosal irritation, along with the chlorine odor side effects that may affect susceptible individuals like those with asthma. Thus, the balance between advantages and disadvantages arising from the application of various types of disinfectants (e.g., sanitizers) is currently a subject of active debate among scientists and clinicians [7,8,9,10].

In Europe and North America, different standards of chlorine-containing products have been adopted. The concentrations of chlorine in commercially available products vary between 4% and 6%, with a suggested concentration of 0.1% sodium hypochlorite for non-healthcare purposes [9,10]. An alternative option is the utilization of 70–90% ethyl alcohol for surface disinfection.

Furthermore, the current situation requires the extensive utilization of household antiseptics such as hand sanitizers for skin hygiene [4]. Hand sanitizers can have the same ingredients as healthcare-grade antiseptics, or they may vary by including additional substances for hydrating and nourishing the skin, flagrant liquids, food dyes (as the safest ones), and other materials. The components that commonly make up hand sanitizers include monohydric alcohols, propylene glycol, amine-containing materials, benzalkonium chloride, and skin care products.

These products are typically categorized as cosmetics, allowing manufacturers to circumvent the need for efficacy testing of their formulations. Currently, the Eurasian Economic Union (EAEU) is developing a project, in which the use of antiseptics in cosmetics is proposed to be limited. However, the regulations in the field of environment protection are becoming stricter all over the world (e.g., [11]), which is an important step towards a significant improvement in the welfare of invertebrates and vertebrates, including humans.

The legislation of the Russian Federation [12] and the European Council Directive 2006/44/EC [13] establishes restrictions for different categories of water use and for water bodies of fishery importance, but the maximum allowable concentrations (MACs) of chemicals in Russia, in contrast with the European Union (EU) regulations, are the same for all categories of water bodies. Some MACs for fishery water bodies in Russia [12] are much stricter. For example, the MAC for Cu established in Russian regulations is significantly lower than the guideline values recommended by the EU Council [13]. The same case is with national environmental quality standards on surfactants, in which MACs can vary from 0.1 to 0.5–0.6 mg/L.

In Russia, environmental regulations do not contain standards or sanitary and hygienic requirements for bottom sediments. Analysis of scientific and methodological literature has shown that there are no unified methods for assessing the state and degree of transformation of technogenically altered soils and sediments, including those under the impact of detergents from herbicide and pesticide formulations.

Thus, the current widespread use (both in the number of persons involved and in amounts) of disinfectants may lead in the future to the uncontrolled release of components into the environment that can negatively affect living organisms and ecosystems.

A number of studies performed on invertebrates showed the impact of surfactants on their different physiological functions and morphological structures e.g., [14,15,16,17]. Surfactant toxicity seems to be mainly related to the alteration of the cellular ionic balance due to changes in the permeability of the cell membrane.

In invertebrates, the test for acute toxicity on the freshwater crustacean *Daphnia magna* Straus, 1820, is highly recommended according to the standard EN ISO 6341 [18]. The essence of the method is finding the concentration of the test substance that causes immobilization of 50% of *Daphnia magna* newborns. This method could be also applicable in the case of studying the effects of surfactants and sanitizers.

The impact of surfactants in combination with other pollutants, such as heavy metals, has not been extensively studied in surface waters. Heavy metals (HMs), such as copper, zinc, lead, cadmium, mercury, arsenic, and more, are high-priority environmental pollutants that demonstrate substantial bioavailability to living organisms. Investigating the factors that influence the bioavailability and penetration of these elements into organisms, as well as the mechanisms and pathways for their excretion from hydrobionts, is an important fundamental objective in the fields of aquatic ecotoxicology and environmental safety [19].

All the factors mentioned above may have a negative impact on the environmental quality and biota as a sensitive component of ecosystems. And the most threatened in such conditions are aquatic invertebrates.

## 3. Use of the Invertebrates in Biomonitoring Studies

Invertebrates are most often and commonly used in biomonitoring studies: about two-thirds of the methods for assessing the quality of flowing waters are based on the use of macrobenthic invertebrates. Among them, mollusks and crustaceans are in priority in biomonitoring [20,21,22,23], as well as in ecotoxicological experiments [24,25,26]. There are several reasons for choosing these macroinvertebrates as bioindicators of environmental quality [27,28,29,30].

Taking into account all the advantages of using these animals, attention should be paid to the fact that ecotoxicological studies and biomonitoring used as routine methods allowing identification of environmental threats often cause “cruel deaths of up to millions of aquatic animals every year” (e.g., [31]). And the data provided by the author of the cited report concern only estimates in relation to Poland.

Invertebrates are “living monitors” of our present and future. It should not be forgotten that due to the fact that mollusks, e.g., from the family Unionidae, are active filter-feeders (one individual filters 20–40 L per day), they maintain the health of the ecosystem through bioaccumulation and biodeposition, participate in the processes of mineralization and, thus, are actively involved in self-purification of water bodies where they live [32]. We agree with J. Mather (2023) that our anthropocentric approach caused “three problems for invertebrates: a lack of knowledge, negative attitudes, and a misunderstanding of their cognitive abilities, all leading to problems for welfare consideration” [33]. Unfortunately, invertebrates are still considered just as convenient and low-cost model objects in ecotoxicology and toxicity research [34].

Consequently, the research is centered on the capacity of mollusks and crustaceans to act as indicators of pollution in coastal waters resulting from the discharge of domestic wastewater containing sanitizers, surfactants, their metabolites, heavy metals, or combinations thereof. At the moment, only a few studies are available on this issue; some of them are discussed below.

## 4. Use of the Invertebrates as Biosensors in Water Quality Monitoring Systems and as Test Organisms in Studying Disinfectants and Surfactants Actions

Invertebrates, mainly mollusks and crustaceans, are successfully used as live monitors of natural surface or drinking water pollution. Automated systems for these purposes have long been actively used in Europe and the United States to monitor water quality, and are called Biological Early Warning Systems (BEWS) [8]. Mussels change their locomotor behavior in response to disturbing agents (e.g., toxins) by closing their shells in order to reduce exposure. Therefore, the frequency of valve opening and closing can be monitored to indicate the avoidance behavior [8]. The commercially available MosselMonitor^®^ system has been used with five different bivalve species (three for freshwater and two for marine water).

In the mentioned BEWS, the heart rate (HR) of mollusks or crustaceans is used as an indicative parameter of current changes in flowing water quality. HR is an integrative measure of the physiological status of the organism, and heart rate variability can serve as a basis for early diagnosis of deterioration at the whole organism level.

Among aquatic invertebrates, mollusks are the most accurate analogs to mammals in terms of the general structure, functioning, and regulatory circuits of cardiac activity. The main parameters of heart rate variability of mollusks are similar to the corresponding values in human rhythmograms [35]. However, studies on the cardiac activity of mollusks and crayfish are quite rare, especially when using automated systems for non-invasive HR monitoring [22,26,36,37,38,39,40,41,42]. Literature on automated systems using mollusks as biosensors is summarized in the review [43].

In early studies, it was shown that crustaceans change rhythms of respiratory and cardiac activities in the presence of HMs [44,45,46,47]. Similar studies were later conducted on crayfish to assess the effects of water disinfection in aquaculture, for example, during its chlorination [48] or chloramination [41]. For example, a 10 mg/L solution of the biocide chloramine-T is commonly used in industry and aquaculture. In experiments on the crayfish *Astacus leptodactylus* (Esch., 1823), the 1-h exposure did not adversely affect the crayfish at chloramine-T concentration of 10 or 50 mg/L [41]. Only after the 1-day exposure at a concentration of 50 mg/L, an obvious toxic effect was noted, leading to significant energy loss in the crayfish.

In the crayfish *Pacifastacus leniusculus*, the effects of ClO_2_ at various concentrations, ranging from 0.01 to 0.29 mg/L, on cardiac activity were studied [48]. The authors noted that in 32% of crayfish individuals, the circadian rhythmicity in the HR is disrupted even at low concentrations of ClO_2_ (0.01–0.2 mg/L), while higher concentrations lead to random periods of tachycardia and bradycardia in all experimental individuals. Crayfish mortality was observed after 3 to 4 weeks of exposure at concentrations exceeding 0.2 mg/L of ClO_2_. The results indicate the importance of maintaining a certain standard of ClO_2_ levels during water treatment, in order to prevent chlorite levels exceeding the WHO guideline value [49].

These studies can serve as a basis for further research on the effects of sanitizers on functional indicators of crustaceans and mollusks. Despite the great interest in the fate of surfactants and disinfectants in freshwater, the effects of environmentally relevant concentrations on target or non-target invertebrates are still unclear.

It is known that a biocenosis responds to significant changes in environmental quality by changing the metabolic rate in the organisms inhabiting it. The efficiency of aerobic energy exchange in organisms, assessed by the rate of oxygen consumption, can serve as an indicator of aquatic environment quality [44,45,46]. Another indicative physiological parameter is the intensity of the filtering activity of mollusks [50,51]. The advantages of using these particular functional indicators, whose changes are linked to the organism’s efforts to prevent negative impact, center around their ability to identify the first signs of pollutants affecting a living organism and to show an early decline in animal health.

It has been noted that synthetic surfactants and detergents have adverse effects on the physiological condition of organisms, the water quality for the hydrobiont welfare, and the self-purification potential of water bodies [32]. The assessment of water pollution is rendered more complex due to the existence of chemical and biological degradation products that are more harmful than the initial compounds [46,47].

Ryabuhina and co-authors [52] reported changes in the dynamics of survival of *Ceriodaphnia* spp. in water samples with detergent. Experiments revealed an increase in the toxicity of sodium dodecyl sulfate (SDS) in a concentration of 25 mg/L after the 15th day of exposure.

There are only a few experimental studies in Russia that have shown the effects of anionic and cationic surfactants, tetradecyl trimethyl ammonium bromide (TDTMA), and SDS, at different concentrations on the antioxidant system (AOS), valve gape, and cardiac activity of the Black Sea mussels (*Mytilus galloprovincialis* Lam.) [53,54,55,56]. Under the action of a cationic detergent TDTMA at a concentration of 0.8 mg/L (a value close to being environmentally relevant) for 8 days, significant changes in the AOS were detected. Marked alterations were observed in the peripheral tissues of mussels, specifically the gills and foot, that were directly exposed to TDTMA [53]. A six-fold increase in the activity of superoxide dismutase (SOD) responsible for O_2_^–^ neutralization was observed in the gills. Concurrently, there was an increase in catalase (CAT) levels by 1.7 and 3.2 times in the gills and foot, respectively. The response of AOS to this surfactant demonstrated tissue specificity, with the hepatopancreas displaying the least sensitivity to the detergent, while the gills exhibited the highest sensitivity to such exposure. The role of AOS in the response of entire organisms to environmental challenges is discussed in [57,58].

Ostroumov (2001) showed that the increase in the SDS concentration in water up to 1.7 mg/L leads to changes in the behavior of the exposed *Mytilus galloprovincialis* with long periods of the mussel being with valves closed [50]. Under these conditions, mussels switch to anaerobic metabolism, and during extended periods of exposure, a condition of oxygen deprivation known as hypoxia may occur. The closure of the mussel’s shell serves as an indication of the adverse impact of the surfactant solution on the mussel [55]. Nonetheless, when exposed to small amounts (0.3–0.5 mg/L) of SDS, mussels detect the presence of this substance in the water, which brings about subsequent alterations in their heart rate and valve movements over time. This underscores the importance of considering the adverse effects associated with low levels of surfactants, which lead to well-expressed disturbances in circadian rhythmicity, with the loss of the predominant activity of mussels at night [56].

Changes in reactive oxidative stress markers were shown by Messina et al. [17] under the action of similar concentrations of SDS (from 0.1 to 1 mg/L), but after a long-term exposure (18 days), in the same mollusk species. Two enzymes, CAT and SOD, involved in reactive oxidative stress have high activity in the digestive gland of the exposed mussels.

Another difficult problem in studying the effects of surfactants and disinfectants is determining the threshold concentrations of the above-mentioned substances. In studies conducted with the use of the SDS, it was shown for the same mollusk species (*Mytilus galloprovincialis* Lam.) from the same water area (Crimea, Sevastopol), that the identified threshold concentration (lowest observed effect concentration—LOEC) can vary significantly, with a difference of up to several times. For example, when studying the inhibition of feeding behavior in the presence of SDS using the photometric method, this value was determined to be 1.7 mg/L [50], and when using non-invasive cardiac activity monitoring it was 0.3–0.5 mg/L [56]. These results raise questions about variables in effective doses that affect the activity patterns of mussels. Therefore, it is highly noteworthy that these levels depend strongly on the sensitivity of the techniques used to measure effects, method of registration, laboratory maintenance of the mussels, and functional system of mussels being tested.

**Figure 2:**
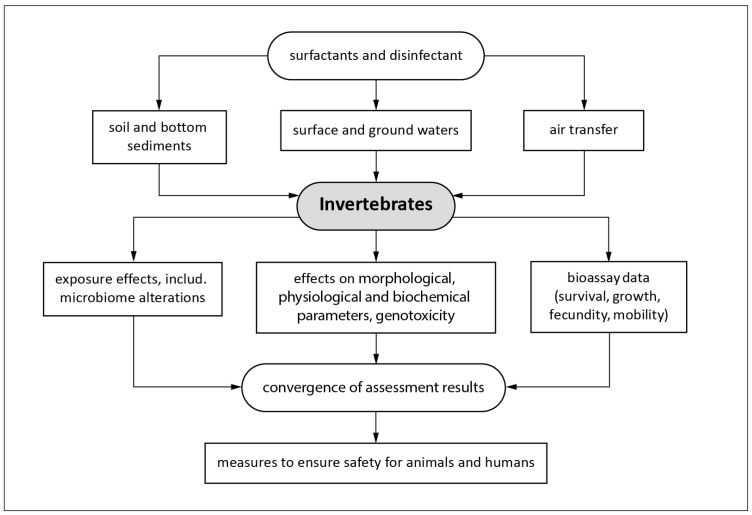
Scheme of the possible pathways of entry of surfactants and disinfectants into the environment and their impact on living organisms.

In the most general approximation, the diagram in Figure 2 shows the entry of the mentioned substances into ecosystems and the impact on invertebrates and vertebrates, including humans, as assessed by the multi-biomarker approach and bioassay methods.

Surface waters are a usual object of environmental studies in terms of heavy metal concentrations, whose maximum allowable levels are set in regulations [6,11]. At the same time, under certain changes in the aquatic environment, HMs from sediments return to the water column via a number of physical, chemical, and biological processes. Thus, bottom sediments are a source of secondary pollution of water systems. Because the chemical behavior and environmental effects of metals in aquatic ecosystems are very complex, studies on HM behavior in sediments have become a hot topic in recent decades [59].

In terms of environmental pollution and its impact on living organisms, HMs in the environment are pollutants of high priority as announced in the Water Frame Directive [60]. The widespread distribution of HMs in the environment significantly endangers environmental safety. They spread far beyond the ranges of their initial emission and, unlike organic ecotoxicants, do not decompose but only change their speciation forms. The HM speciation forms are diverse and differ in toxicity. At the same time, “free” uncomplexed HM cations are considered to be the most toxic and the bound heavy metals are considered less dangerous because they lose bioavailability [61,62].

The main natural processes affecting the transformation and migration of HM compounds, including the most toxic labile forms, are the sorption of HM ions by dispersed minerals and complexation with humic acids. Therefore, to assess and predict the occurrence of environmental threats associated with the formation of labile HM forms in water bodies, it is necessary to study the basic regularities of metal ions binding to these materials. In this case, two main processes should be taken into account: the chemical equilibration of HM forms and the kinetics of speciation (sorption, complexation, etc.).

In a number of studies, the distribution of HMs, their accumulation in various ecosystems, in organs of living organisms, both aquatic and terrestrial, have been discussed [61,62,63]. However, it should be noted that in some species of crustaceans toxico-resistance to HM was observed [64]. One of the recently published studies is a valuable review based on observations in crustaceans [65]. It contains data on the elemental composition of HMs in the environment, their bioaccumulation in different animals’ tissues, routes of excretion from the body, enzymatic responses, etc. In all models of biogeochemical cycles of pollutants that are created to assess hazards, it is necessary to take into account the interaction of pollutants with humic acids, since it radically changes both the chemical and toxicological behavior of harmful materials [66].

Another, no less important natural sorbent of HMs is finely dispersed minerals. A certain part of finely dispersed mineral suspensions consists of nanominerals, whose particles are of the nanometer size. Scientific studies raise concerns about the rapidly increasing production and consumption of nanomaterials, which will inevitably lead to their accumulation in the environment. The unique properties of nanoparticles, such as their huge surface area and abundance of surface reaction sites, facilitate their interaction with various environmental contaminants and toxicants, posing a new environmental threat [62,66].

The ability of sediments to accumulate ecotoxicants is influenced by physical, chemical, and biological factors. The physical factors include, first and foremost, the granulometric composition of sediments. Finely dispersed particles in sediments play a huge role in interfacial interactions, ion exchange, adsorption, structure formation, and immobilization of HMs; and studying the particle size distribution in relation to the sorption properties of sediments is of paramount importance [62,67,68,69].

At the same time, it is crucial to investigate the impact of biota and their metabolites on the fate of HMs in the environment [65]. It is worth mentioning that only labile hydrated ions or unstable complexes can easily cross cell membranes and are therefore biologically active. The most accessible forms of metals for benthic organisms are those dissolved in the pore water of sediments [70]. A study [71] explored how Cd^2+^ and Cu^2+^ ions affect the sorption of atrazine, a common herbicide, by bottom sediments. The research revealed that Cd enhances the sorption of atrazine synergistically, while Cu has an antagonistic effect. Therefore, understanding the patterns of the mutual influence of these toxicants on sorption processes seems necessary and very relevant. 

Many studies have shown that heavy metal ions can accumulate in living organisms, disrupting their metabolic processes and inhibiting the production of proteins, e.g., enzymes [19,63,65]. Summarizing, we need to stress that all measurements, limitations, and regulations should aim at ensuring the health of the organisms and their environment. And, first of all, this concerns invertebrate animals, as sentinel species, and their welfare.

In assessing the biological impacts of heavy metal pollution on the environment, a common practice is to measure the bioconcentration factor (BCF) of heavy metals in animal tissues [72]. The accumulation of HMs in tissues, particularly for copper, zinc, lead, and cadmium, displays tissue specificity. Moreover, differential sensitivity to metals has been observed in mussel tissues. (e.g., [73,74,75,76]). BCF values of Cd, Cu, Pb, and Zn exceeding 1000 in mussels indicate that mussels have a great capacity to accumulate these metals. Thus, the mussels can transfer these metals to higher trophic levels, ultimately posing a potential hazard to human health.

In a number of studies, different biological effects of HMs on the physiological and biochemical indicators of aquatic organisms’ state and mechanisms of organisms’ adaptations were demonstrated [62,75,76]. etc. Most of these studies were carried out on bivalves and crustaceans, both of marine and freshwater origin, e.g., [64,65,75,76].

The ability of macrobenthic invertebrates, including mollusks and crustaceans, to absorb HMs depends on the metal speciation and the organism’s characteristics. Therefore, it is essential to consider organisms’ bioaccumulation capacity alongside data on metal concentrations and speciation forms in the abiotic parts of the ecosystem [77]. Voltammetry results indicate that the amounts of HMs, such as Cu, Cd, Zn, and Pb, that are readily bioavailable to these organisms can be affected by the pH of the aqueous solution used in the experiment. In the case of natural waters, the presence of disinfectants (sanitizers) and surfactants in water can change pH, thereby significantly affecting the solubility of other organic materials and increasing the number of labile forms of metals in the aquatic environment.

Summarizing, the accumulation of detergents, sanitizers, or their metabolites together with other environmental pollutants in the aquatic environment can disrupt the environmental safety/welfare of terrestrial and aquatic biota. Representatives of invertebrates play a key role in different ecosystems as environmental engineers (bioconstructors) [78]. In the presence of detergents that significantly change the pH of the environment, these animals may experience disturbances in the functioning of mucous structures and, consequently, excretory functions (for example, the production of secretions and feces). This disrupts building their tube-dwellings and producing similar materials for other animals in their common ecosystem [78]. Therefore, further research is needed on the effects of detergents and disinfectants on the essential functional systems of invertebrates.

## 5. Possible Ways to Reduce Risks for Animals and Humans

The current EU strategy on the protection of animals includes the implementation of the concept called “3Rs”, i.e., reducing the number of animals in experiments (reduction), reducing the deprivation in the course of the experiments (refinement) and the ultimate aim is to completely replace testing on vertebrate animals (replacement) by alternative in vitro methods in the practice of research facilities. Yet, no specific invertebrate species are mentioned there.

One of the possible ways to reduce the intake of emerging substances toxic to animals (in particular, aquatic invertebrates) and humans may be seeking, replacing and further using disinfectants and surfactants that are less hazardous in their effects on biota. For example, according to the Centers for Disease Control and Prevention (CDC), vigorous handwashing in warm water with plain soap for at least 20 s is sufficient to fight germs in most cases. When plain soap and water are not available, the use of an alcohol-based hand sanitizer product is a better option than soap containing triclosan which is widely used as a disinfectant in households.

Another approach is the use of a variety of different test organisms from different trophic levels to ensure the negative or positive effects of disinfectants on living organisms. This approach is highly recommended by the Baltic Marine Environment Protection Commission (Helsinki Commission, HELCOM) for studying the toxicity of individual substances or their mixtures on benthic invertebrates [79].

It is necessary to note that the introduction of new advanced methods can significantly reduce freshwater invertebrate mortality and help in a more thorough assessment of the negative effects of toxicants (see SETAC Europe, 2022, discussion [80]). This concerns, for example, non-invasive (non-destructive) assessment methods, namely recording certain physiological functions (respiration, oxygen consumption, cardiac activity, and valve or gill movements) in mussels, clams, and crustaceans, where possible. Development of these assessment methods and techniques for their better performance can also become an important step towards a significant improvement in the welfare of aquatic invertebrates.

At present, the recommendations for disinfectant/detergent disposal routes differ according to the regionally established disposal structures. For example, according to Recommendations of the German Environment Agency disinfection of wastewater is accomplished both by filtering out harmful microorganisms and by adding disinfectant chemicals [80]. Water is disinfected so as to kill any pathogens that pass through filters and to provide a residual dose of disinfectant to kill or inactivate potentially harmful microorganisms in the storage and distribution systems.

In nowadays practice, chlorine dioxide ClO_2_ is relatively rarely used in disinfection, because in some circumstances it may create excessive amounts of chlorite, whose release is strictly regulated, e.g., in the United States, to be at the lowest possible levels [11,50].

While a large proportion of the substances used in detergent formulations are subject to compulsory registration under the Registration, Evaluation, Authorization, and Restriction of Chemicals (REACH), there is still often too little or nothing known about their environmental properties. Accordingly, further investigations on levels of ingredients from detergent formulations in water are needed. First, the problematic ingredients relevant to monitoring should be identified. This requires, among other things, information on their toxicological relevance. In order to prioritize substances in the future in terms of monitoring and possible restrictions, the Germany Protection Agency—Umweltbundesamt (UBA)—is currently developing its own public information system, which will provide the necessary information about detergent ingredients by the end of 2024 at the latest [81].

Creating and improving evaluation bases and criteria for pollutants is of importance on a worldwide scale. Research into the entry of poorly biodegradable substances from detergents into waters remains one of the most important monitoring programs. There is a need for discussion on the restriction of poorly degradable substances and the development of analytical methods as a prerequisite for targeted monitoring.

One of the possible ways to reduce the content of disinfectants and detergents in the environment is phasing out the agricultural use of sewage sludge. The recommendations for sludge disposal routes differ according to the regionally established disposal structures [81].

Another demand is using potential synergies between the EU Council directives that provide measures to reduce the emission of disinfectants and detergents and their by-products in the environment.

Moreover, information on the effective use of disinfectants and surfactants, applied doses, and rules on disposal and storage of hazardous wastes should be disseminated among the population and attract social attention.

## 6. Conclusions

In the face of new challenges associated with the SARS-CoV-2 pandemic, greater attention should be paid to the compromise between harm and benefit in the application of disinfectants and sanitizers in our lives. It is necessary to take a closer look at the possible negative effects of these substances on the health of the Invertebrates already suffering from environmental pollution. Under development are new non-invasive methods of testing possible negative effects of these substances and their combined effects with high-priority pollutants (from the List of HELCOM) on organisms, especially aquatic invertebrates (as the main target species) of known contaminants or recently synthesized harmful substances, which could pose threats to animals’ welfare.

Perhaps, there will be further proposals on this issue to ensure the welfare of the Invertebrates in light of the novel coronavirus pandemic and its associated challenges.
